# 

**Published:** 1866-10

**Authors:** 


### CONTENTS FOR OCTOBER.

ORIGINAL CONTRIBUTIONS.

Clinical Lectures on Diseases of the Eye. By E. L. Holmes, M. D...p. 443

Twins, with One Placenta, Two Cords and One Set of Membranes. By S. A. Ferrin, M. D. .... 449

The Recent Epidemic of Cholera at the County Hospital. Reported by T. Bevan, M.D .... 450

A New Remedy in Erysipelas. By H. B. Withers, M.D. .... 459

Aneurism of the Abdominal Aorta—Ligature of the Right Common Iliac. By A. J. Baxter, M.D .... 460

SELECTED ARTICLES.

Cholera on Blackwell's Island .... 463

Prevention of Cholera in New York .... 465

Remedies for Diarrhœa .... 466

Sympathy between the Auditory Canal and the Larynx .... 471

Contributions for the Sanitary Commission Volume .... 472

Physiological Action of Narceine .... 473

Armenian or Diamond Cement .... 473

Bromide of Potassium in Sleeplessness consequent upon Uterine Irritation .... 474

Decision against River Pollution .... 474

PROCEEDINGS OF MEDICAL SOCIETIES.

Chicago Medical Society .... 475

Sanitary Condition of the City—Opium in Cholera Infantum—New Anæsthetic—Acute Conjunctivitis—Pressure in Conjunctivitis.

EDITORIAL .... 480

*Book Notices*—Medical Electricity—A Treatise on the Origin, Nature, Prevention and Treatment of Asiatic Cholera—The Principles of Surgery—On Spermatorrhœa—A Manual of the Principles of Surgery, based on Pathology, for Students—The Medical Register of the City of New York for 1866.

Pamphlets Received .... 487

Chicago Hospital for Women and Children .... 487

The Order of the Police in reference to the Privileges of Physicians in Crossing Chicago Bridges .... 489

California Wines and other Notices .... 490

